# Presence or Emergence of Canine Leishmaniasis, Malawi

**DOI:** 10.3201/eid3201.250855

**Published:** 2026-01

**Authors:** Boniface Chikufenji, Kyoko Hayashida, Yasuyuki Goto, Tatsuki Sugi, Chizu Sanjoba, Chrispin Njala, Inga McDermott, Frederic Lohr, Dagmar Mayer, Shohei Ogata, Masahiro Kajihara, Naganori Nao, Ryo Nakao, Laston Chimaliro, Donales Kapira, Janelisa Musaya, Junya Yamagishi, Elisha Chatanga

**Affiliations:** Ministry of Agriculture, Lilongwe, Malawi (B. Chikufenji); Hokkaido University, Sapporo, Japan (B. Chikufenji, K. Hayashida, T. Sugi, S. Ogata, M. Kajihara, N. Nao, R. Nakao, L. Chimaliro, J. Yamagishi); University of Zambia, Lusaka, Zambia (K. Hayashida, S. Ogata, M. Kajihara); University of Tokyo, Tokyo, Japan (Y. Goto, C. Sanjoba); Mission Rabies Malawi, Blantyre, Malawi (C. Njala, I. McDermott, F. Lohr, D. Mayer); Malawi–Liverpool Wellcome Programme, Blantyre (D. Kapira, J. Musaya); Lilongwe University of Agriculture and Natural Resources, Lilongwe (E. Chatanga)

**Keywords:** leishmaniasis, parasites, vector-borne infections, canine, Leishmania infantum, Malawi, neglected tropical diseases, zoonoses

## Abstract

Canine leishmaniasis has long been thought to be absent in Malawi. However, our cross-sectional study in indigenous dogs showed a high prevalence of *Leishmania* infection in some areas, where seropositivity rates reached up to 7.0%. These findings suggest that this neglected zoonotic disease may already be endemic in Malawi.

Leishmaniasis is a neglected tropical diseases that is transmitted by female phlebotomine sandflies and affects >1 million persons annually (*1*). The disease manifests in 3 clinical forms, cutaneous, mucocutaneous, and visceral. Of those, human visceral leishmaniasis represents the most severe manifestation and is the second-leading cause of parasitic disease–related deaths in humans after malaria, causing >30,000 deaths annually (*1*). Infection with *Leishmania infantum* in humans causes severe systemic disease, with a higher risk for progression in infants and HIV-infected patients (*1*).

Dogs are the primary reservoirs of *L. infantum*, making them critical targets for surveillance and control to reduce the risk for zoonotic leishmaniasis. Although canine leishmaniasis, which is caused by *L. infantum*, has been widely reported in subtropical and tropical regions, including Asia, North Africa, Southern Europe, and the Americas (*2*), recent data from sub-Saharan Africa remain scarce. Human cutaneous leishmaniasis, which is caused by an unidentified *Leishmania* species, was reported in Malawi in 1993 (*3*), and 1 seropositive dog was reported in 2016 (*4*), but to our knowledge, no data regarding *Leishmania* infections in humans or animals have been reported since. 

In Zambia, a neighboring country of Malawi, canine leishmaniasis was first reported in 1994 (*5*). Subsequently, no further cases were reported for nearly 30 years, until a 2021 study identified autochthonous infection cases of canine leishmaniasis in 3 dogs (*6*). Furthermore, a recent study conducted in 2022 revealed a high seroprevalence of *Leishmania* antibodies in dogs from 2 urban cities in Zambia (*7*). The reported high seroprevalence of *Leishmania* infection in Zambian dogs (≈17%) prompted us to investigate the presence of *Leishmania* infection in dogs in Malawi.

## The Study

**Figure 1:**
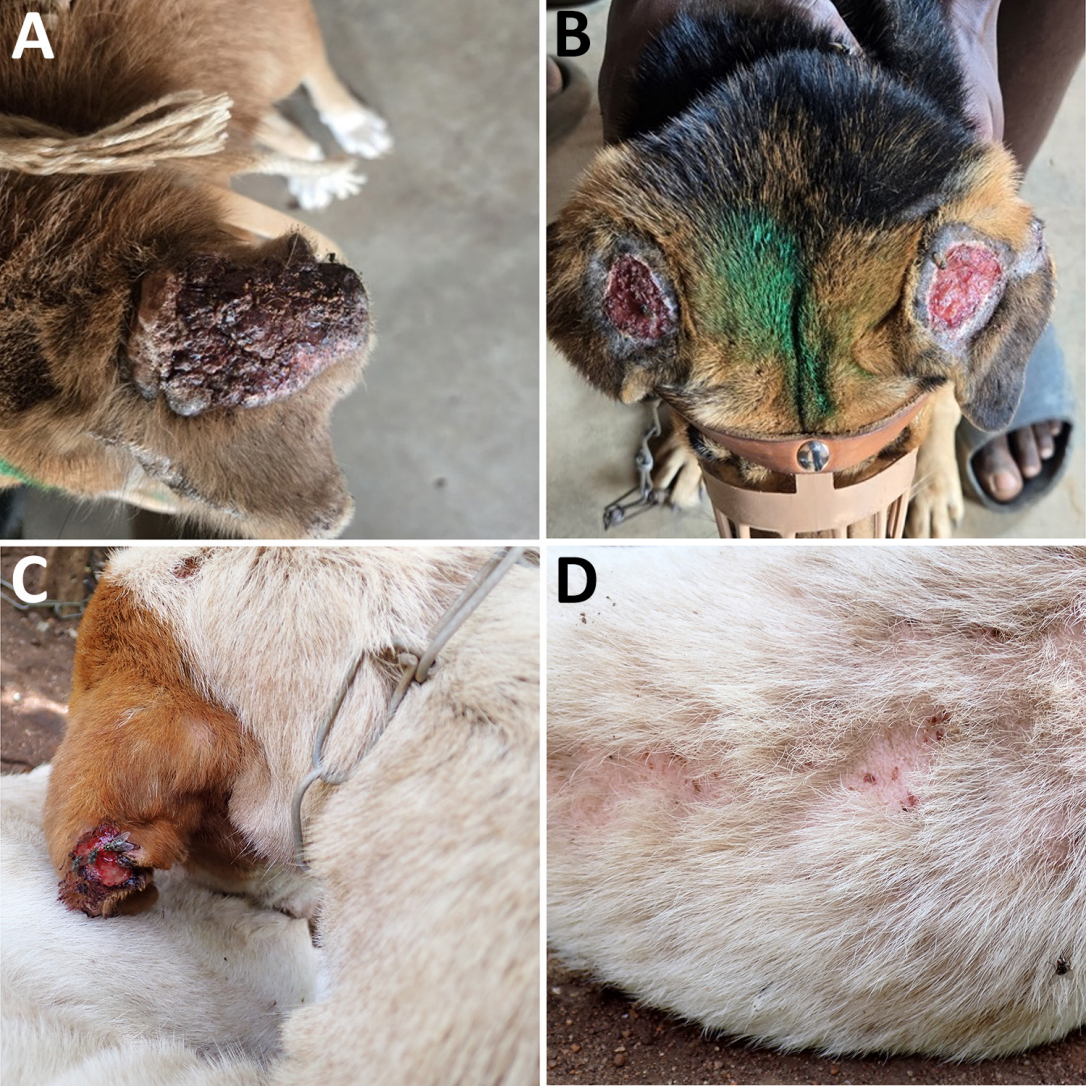
Clinical manifestations of *Leishmania infantum*‒infected dogs in Mchinji, Nkhotakota, and Zomba districts in a study of canine leishmaniasis, Malawi. A) Severely crusted, thickened dark surface pinna (24MWD_C073). B) Bilateral ulcerative lesions on the ears with an exposed tissue (24MWD_C110). C, D) Unilateral ulcerative lesions on the ears and alopecia with severe thinning of the fur (24MWD_C040).

During 2023–2024, we collected 557 canine blood samples through convenience sampling during mass rabies vaccination campaigns, including 157 samples from Mchinji District, 100 from Nkhotakota District, and 300 from Zomba District (Table, Appendix 1 Figure 1). The areas of sampling were semiurban or rural, and all dogs were nonpedigreed, local, or mixed breeds based on visual observations. Most of the dogs were semi–free-ranging and lived outdoors. We took records and photographs for dogs showing alopecia, emaciation, or other abnormalities (Figure 1; Appendix 2). We obtained blood from each dog into an EDTA tube and stored the samples in a DNA/RNA shield (Zymo Research, https://www.zymoresearch.com). We also obtained plasma from the remaining blood. The study protocol was approved by the Department of Animal Health and Livestock Development in Malawi (approval no. DAHLD/AHC/07/2024/01).

**Table:** PCR and ELISA results for detecting *Leishmania* spp. in indigenous dogs from the Mchinji, Nkhotakota, and Zomba districts in a study of canine leishmaniasis, Malawi*

District	qPCR-positive†	SSU–cPCR-positive	ITS2–cPCR-positive	LdSLA-seropositive	rK39r4-seropositive	PCR-positive‡	Serologic test–positive§	Both PCR and serologic test–positive
Mchinji	12/157 (7.6)	17/157 (10.8)	13/157 (8.3)	0/140 (0)	1/140 (0.7)	17/157 (10.8)	1/140 (0.7)	0/140 (0)
Nkhotakota	5/100 (5.0)	5/100 (5.0)	5/100 (5.0)	0/98 (0)	0/98 (0)	5/100 (5.0)	0/98 (0)	0/98 (0)
Zomba	9/300 (3.0)	22/300 (7.3)	17/300 (5.7)	16/300 (5.3)	15/300 (5.0)	23/300 (7.7)	21/300 (7.0)	10/300 (3.3)
Total	26/557 (4.7)	44/557 (7.9)	35/557 (6.3)	16/538 (3.0)	16/538 (3.0)	45/557 (8.4)	22/538 (4.1)	10/538 (1.9)

*All values are no. (%). cPCR, conventional PCR; ITS2, internal transcribed spacer 2; LdSLA, *Leishmania donovani* soluble lysate antigen from cultured promastigotes; qPCR, real-time PCR; rK39r4, recombinant rK39r4 antigen; SSU, small subunit. †Defined as cycle threshold value <35. ‡Defined as the count of the sample that showed positives in any of the 3 tests: qPCR, SSU-cPCR, or ITS2-cPCR. §Defined as the count of the sample that showed positives in LdSLA, rK39r4 antigen, or both.

**Figure 2:**
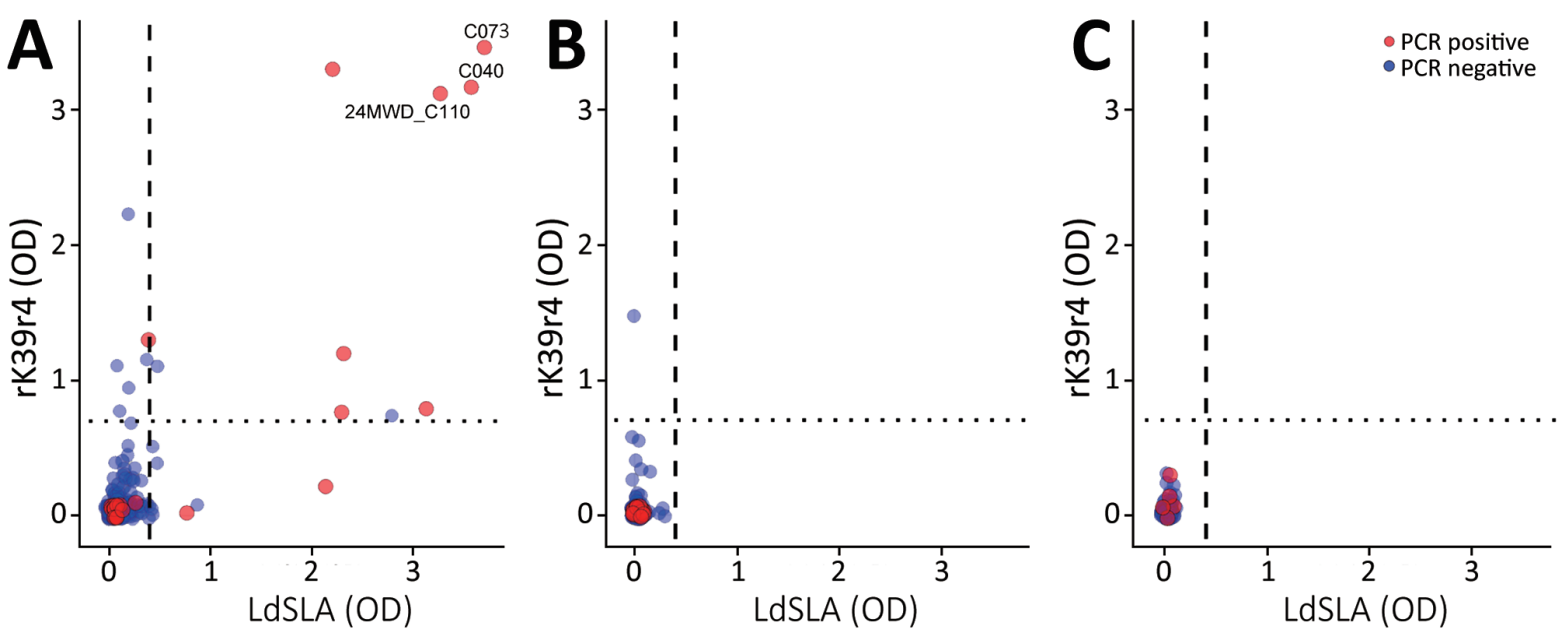
Scatterplots showing ELISA OD values for LdSLA and rK39r4 in indigenous dogs from Mchinji, Nkhotakota, and Zomba districts in a study of canine leishmaniasis, Malawi (Zomba, 300 samples; Mchinji, 140 samples; and Nkhotakota, 98 samples). Red indicates PCR-positive samples. Identification numbers of the dogs that exhibited clear clinical manifestations are also shown, corresponding to numbers in Figure 1. Plots were generated by using R studio software version 4.4.1 (https://rstudio.com/products/rstudio). Cutoff OD values (0.4 for LdSLA, vertical dashed line; 0.7 for rK39r4, horizontal dotted line) were determined as mean of the healthy endemic control dogs plus 5 SDs. LdSLA, *Leishmania donovani* soluble lysate antigen from cultured promastigotes; OD, optical density; rK39r4, recombinant rK39r4 antigen.

We extracted DNA by using NucleoSpin DNA Blood Extraction Kit (Macherey-Nagel, https://www.mn-net.com) and amplified it by using conventional PCR targeting the small subunit RNA region (*8*) and internal transcribed spacer 2 region (*9*). We also tested DNA by using real-time PCR targeting kinetoplast DNA (*10*). The PCR detection targeting the small subunit region showed an overall detection rate of 7.9% (44/557) (Table). Similarly, PCR targeting the internal transcribed spacer 2 region identified an overall detection rate of 6.3% (35/557) (Table). Real-time PCR analysis demonstrated a detection rate of 4.7% (26/557) (Table). In total, 8.1% (45/557) of the samples showed >1 positive results in 3 PCR tests (Table). We sequenced positive samples from conventional PCR and confirmed that they were identical to the *L. infantum* reference genome (JPCM5 strain, GenBank accession no. GCF_000002875) (Appendix 2). In addition, we amplified the entire internal transcribed spacer region sequences (*11*) from the representative sequences in each district and compared them with global *L. infantum* sequences. As a result, all sequences from Malawi and most *L. infantum* sequence strains clustered together, corresponding to *L. infantum* zymodeme MON-1 (Appendix 1 Figure 2), the predominant zymodeme found worldwide in both humans and dogs, suggesting a shared ancestry of the parasite.

We also conducted ELISA for detecting IgG (*12*) by using *L. donovani* soluble lysate antigen from cultured promastigotes (LdSLA) and recombinant rK39r4 antigens. We used serum from clinically confirmed canine leishmaniasis dog (*6*) as a positive control, and we used healthy endemic dog control serum samples from 18 healthy confined breeding dogs in Malawi to determine the optimal cutoff value (mean +5 SD). As a result, we observed a 3.0% (16/538) seropositivity for each antigen and an overall seroprevalence of 4.1% (22/538) (Table; Figure 2). The results of PCR and ELISA were not entirely concordant, and we found a large number of PCR-positive dogs to be seronegative (Figure 2). However, dogs exhibiting double seropositivity (i.e., positive on PCR and ELISA) for LdSLA and rK39r4 antigens were significantly more likely to be PCR-positive (odds ratio 57.1, 95% CI 13.7–387.4). In addition, dogs showing severe clinical signs were mostly positive by both PCR and ELISA (Figure 1, Figure 2).

**Figure 3:**
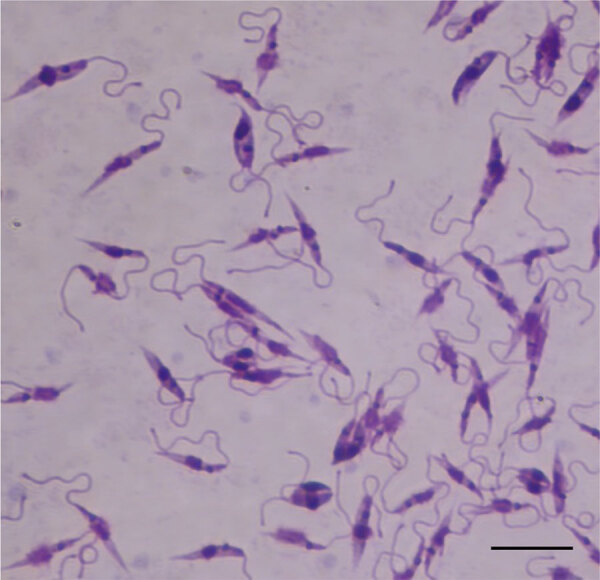
Promastigote stage of *Leishmania infantum* isolated from dog in Zomba District (24MWD_C040) cultured in Novy-MacNeal-Nicolle medium in a study of canine leishmaniasis, Malawi. The elongated, flagellated forms characteristic of extracellular stage observed under light microscopy. Giemsa stain. Oil immersion. Scale bar indicates 10 µm.

After observing high seropositivity in Zomba District, we followed up on some of the seropositive dogs. We performed the original sample collection in September 2024 and revisited the area in March 2025. We found that 1 dog (24MWD_C040) was seriously ill (Figure 1), and we performed fine-needle biopsy on the enlarged superficial cervical lymph node. We cultured the collected fluid at room temperature in Novy-MacNeal-Nicolle medium overlaid with M199 media (*13*). Eleven days after inoculation, we observed a motile promastigote stage of the parasite and confirmed the internal transcribed spacer sequence to be *L. infantum*, further confirming the presence of the parasite (Figure 3; Appendix 1 Figure 2).

## Conclusions

This study confirmed the presence and high endemicity of *L. infantum* in indigenous dogs in Malawi. Our study found that both symptomatic and asymptomatic dogs tested positive for *L. infantum* DNA. In addition, we successfully isolated the promastigote stage of the parasite from 1 dog, which showed severe clinical manifestations, was positive in all PCRs, and had high ELISA optical density values for LdSLA and rK39r4. This finding suggests that double positivity by PCR and ELISA is an indicator of disease progression, whereas PCR or ELISA single-positive dogs might still be asymptomatic carriers. As such, the strategic use of LdSLA and rK39r4 antigens in ELISA and PCR for *L. infantum* testing will enhance diagnostic performance by increasing the tests’ ability to correctly identify infected dogs. 

Zomba District showed the highest disease prevalence in ELISA positives and PCR–ELISA double positives. Compared with the districts of Nkhotakota and Mchinji, Zomba can be characterized as urban, where higher dog-to-human population densities are observed. Because of the lack of information about vector sandfly distribution in Malawi, speculating on the possible risk factors explaining this prevalence difference by the region is difficult. Nevertheless, a higher prevalence in densely populated areas also has been reported in Zambia and Brazil (*7*,*14*). Canine leishmaniasis has been documented as a precursor to human outbreaks in other regions (*15*). 

In summary, we report a high prevalence of *L. infantum* in dogs from 3 districts in Malawi, as detected by molecular methods and serologic assays. A live parasite also was confirmed and isolated from the lymph node biopsy fluid of 1 dog. Our findings indicate the possible emergence or reemergence of the disease in the country, highlighting the urgent need for broader disease surveillance of the disease in humans, dogs, and the sandfly vector. Our results underscore the role of dogs as reservoirs of *L. infantum* in Malawi, posing a zoonotic risk, and highlight the need for urgent public health interventions to prevent its spread.

Appendix 1 Additional information about presence or emergence of canine leishmaniasis, Malawi.

Appendix 2 Demographic data and laboratory test results for dogs sampled in study ofpresence or emergence of canine leishmaniasis, Malawi (.
