# Supplementary material for: Presence or Emergence of Canine Leishmaniasis, Malawi
Source: Emerg Infect Dis. 2026 Jan;32(1):137–41. doi: 10.3201/eid3201.250855 (PMC12870074; doi:10.3201/eid3201.250855)
Supplement: Appendix 1 — Additional information about presence or emergence of canine leishmaniasis, Malawi. [file 25-0855-Techapp-s1.pdf]

Article DOI: https://doi.org/10.3201/eid3201.250855

EID cannot ensure accessibility for supplementary materials supplied by authors. Readers who have difficulty accessing supplementary content should contact the authors for assistance.

Presence or Emergence of Canine Leishmaniasis, Malawi

Appendix 1

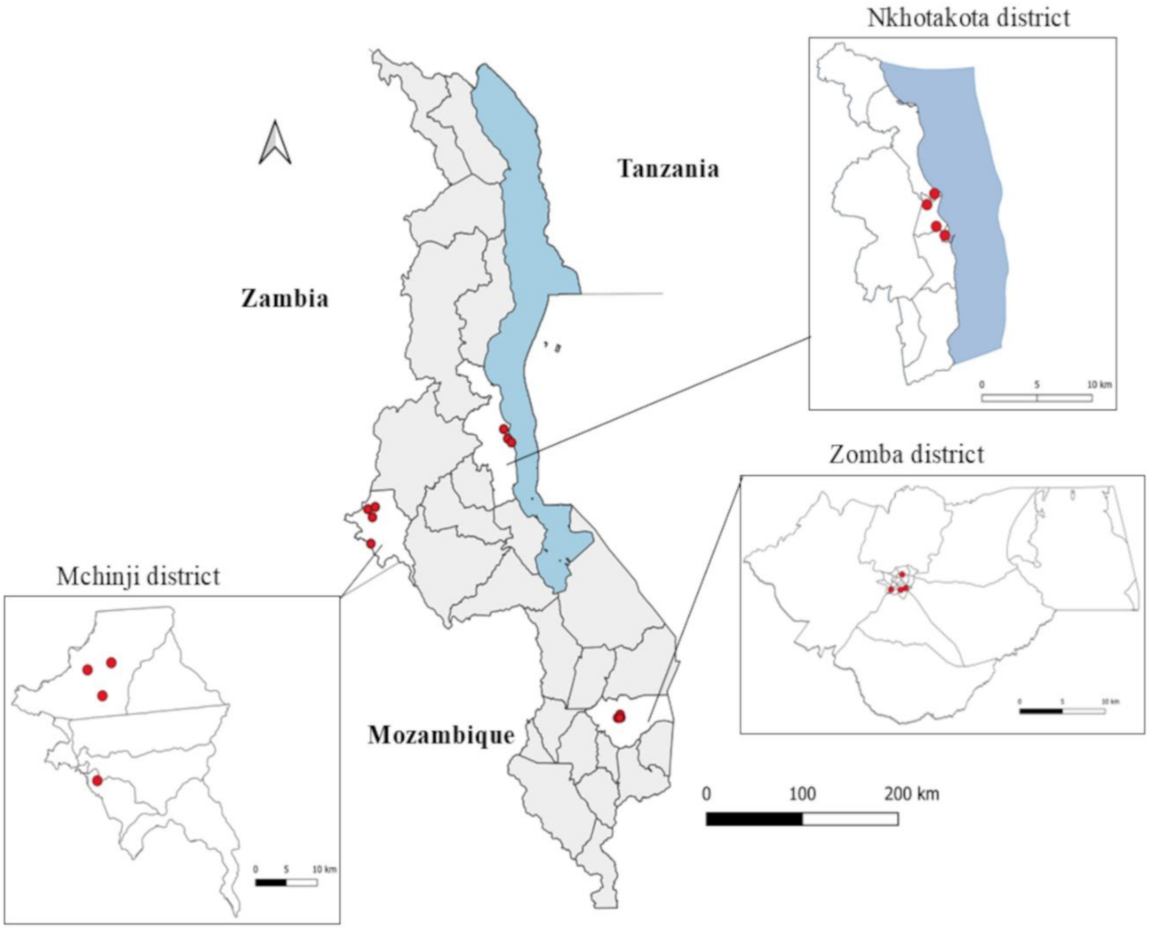

Appendix 1 Figure 1. Map of Malawi showing sampling sites. The map was generated using QGIS software (version 3.40.1).

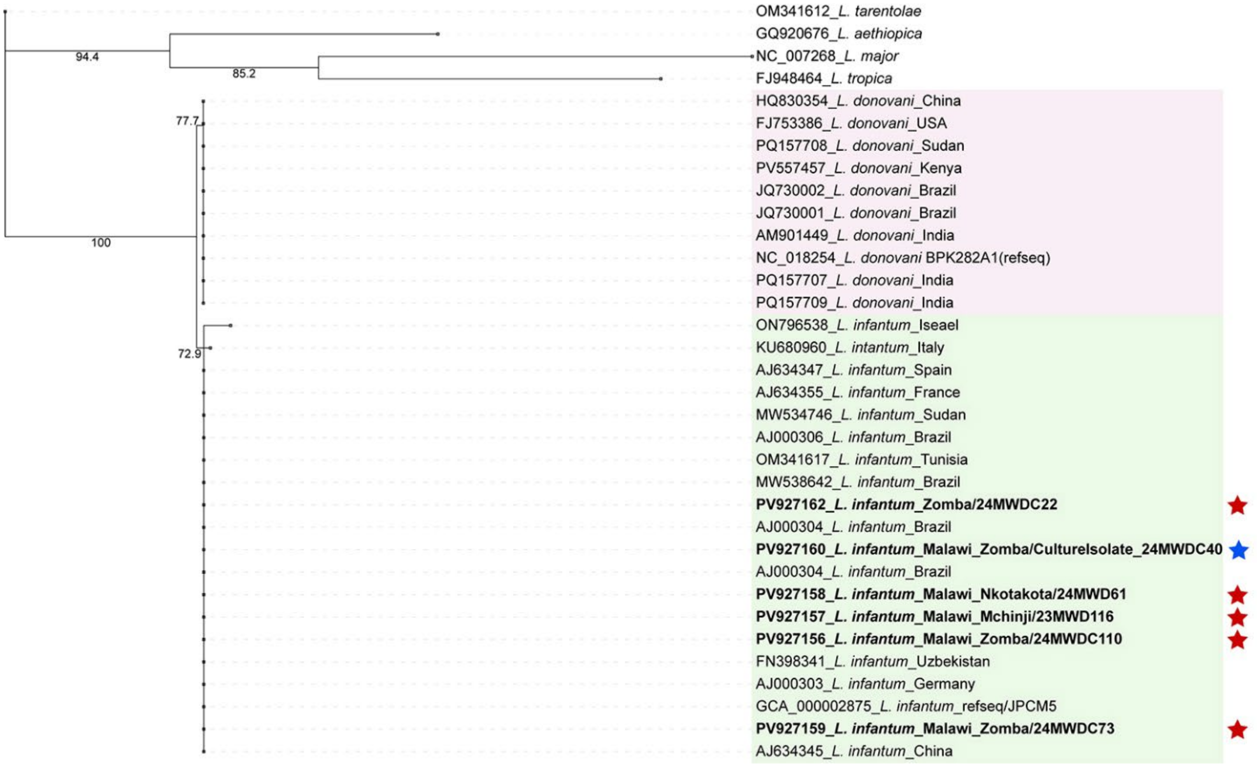

Appendix 1 Figure 2. Maximum likelihood phylogenetic tree showing the relationship between entire ITS sequences (ITS1, 5.8S, ITS2) of the study (bold) and selected *L. infantum* sequences obtained from NCBI GenBank. Red stars: sequences obtained from blood samples. Blue star: sequence obtained from an in vitro cultured isolate from Zomba dog 24MWD_C040. Sequences were aligned using MAFFT with L-INS-i method. After manual trimming, a maximum likelihood tree was conducted using IQ-TREE 3 using TPM2u+F+I, which was selected by ModelFinder Plus. All bootstrap values greater than 75% from 1000 replications are shown on the branch nodes. *Leishmania tarentolae* (OM341612) was used as the outgroup.
